# Supplementary material for: Six years of measuring patient experiences in Belgium: Limited improvement and lack of association with improvement strategies
Source: PLoS One. 2020 Nov 3;15(11):e0241408. doi: 10.1371/journal.pone.0241408 (PMC7608918; doi:10.1371/journal.pone.0241408)
Supplement: S1 Table — (DOCX) [file pone.0241408.s001.docx]

**S1 Table. Trends in patient experience scores across Flemish acute-care hospitals (n=44).**

|  |  | **2014** | | **2015** | | **2016** | | **2017** | | **2018** | | **2019** | |
| --- | --- | --- | --- | --- | --- | --- | --- | --- | --- | --- | --- | --- | --- |
| **Dimension of patient experience (Question)^(1)^** | **Linear trend β^(2)^ (95% CI)** | **Top-box score (%)** | **β^(3)^  (95% CI)** | **Top-box score (%)** | **β^(3)^  (95% CI)** | **Top-box score (%)** | **β^(3)^  (95% CI)** | **Top-box score (%)** | **β^(3)^  (95% CI)** | **Top-box score (%)** | **β^(3)^  (95% CI)** | **Top-box score (%)** | **β^(3)^  (95% CI)** |
| Preparing for hospital stay | 0.57 | 76 | 0 | 75 | -1.19 | 76 | -0.13 | 77 | 1.24 | 77 | 0.65 | 78 | 2.46 |
|  | (0.31; 0.82)** |  | / |  | (-2.67; 0.29) |  | (-1.61; 1.35) |  | (-0.25; 2.73) |  | (-0.85; 2.15) |  | (0.94; 3.97)** |
| Information about condition | 0.50 | 51 | 0 | 50 | -0.40 | 51 | -0.15 | 51 | 0.32 | 52 | 1.06 | 53 | 2.45 |
|  | (0.28; 0.71)** |  | / |  | (-1.66; 0.86) |  | (-1.41; 1.11) |  | (-0.95; 1.59) |  | (-0.21; 2.34) |  | (1.16; 3.74)** |
| Information about treatment and procedures | 0.29 | 54 | 0 | 52 | -2.29 | 52 | -2.78 | 53 | -1.64 | 54 | -0.35 | 55 | 0.41 |
|  | (0.06; 0.52)* |  | / |  | (-3.54; -1.03)** |  | (-4.03; -1.52)** |  | (-2.90; -0.37)* |  | (-1.62; 0.93) |  | (-0.87; 1.70) |
| Dealing with patients and collaboration between healthcare providers | 0.30 | 76 | 0 | 76 | 0.06 | 76 | -0.17 | 77 | 0.42 | 77 | 1.14 | 78 | 1.30 |
|  | (0.13; 0.47)** |  | / |  | (-0.96; 1.09) |  | (-1.19; 0.86) |  | (-0.61; 1.45) |  | (0.10; 2.18)* |  | (0.25; 2.35)* |
| Privacy | 0.46 | 80 | 0 | 80 | -0.08 | 81 | 0.56 | 81 | 1.09 | 82 | 1.78 | 82 | 1.94 |
|  | (0.26; 0.65)** |  | / |  | (-1.24; 1.08) |  | (-0.60; 1.72) |  | (-0.08; 2.25) |  | (0.60; 2.95)** |  | (0.75; 3.12)** |
| Safe care | 2.65 | 52 | 0 | 53 | 0.57 | 56 | 4.06 | 61 | 8.65 | 62 | 10.16 | 64 | 11.69 |
|  | (2.37; 2.94)** |  | / |  | (-1.05; 2.18) |  | (2.44; 5.67)** |  | (7.02; 10.27)** |  | (8.52; 11.80)** |  | (10.03; 13.34)** |
| Pain management | 0.60 | 75 | 0 | 74 | -1.33 | 75 | -0.07 | 76 | 1.08 | 77 | 1.52 | 77 | 2.07 |
|  | (0.39; 0.80)** |  | / |  | (-2.52; -0.13)* |  | (-1.26; 1.12) |  | (-0.12; 2.28) |  | (0.31; 2.72)* |  | (0.85; 3.29)** |
| Discharge | -0.09 | 89 | 0 | 88 | -0.69 | 88 | -1.16 | 88 | -0.92 | 88 | -0.82 | 88 | -0.63 |
|  | (-0.18; 0.01) |  | / |  | (-1.23; -0.14)* |  | (-1.70; -0.62)** |  | (-1.47; -0.37)** |  | (-1.37; -0.27)** |  | (-1.19; -0.08)* |
| Global (Rating the Hospital) | 1.10 | 56 | 0 | 56 | 0.00 | 57 | 1.15 | 58 | 2.30 | 60 | 3.65 | 61 | 5.19 |
|  | (0.80; 1.40)** |  | / |  | (-1.77; 1.78) |  | (-0.63; 2.93) |  | (0.51; 4.09)* |  | (1.84; 5.45)** |  | (3.36; 7.01)** |
| Global (Recommending the hospital) | 0.39 | 67 | 0 | 68 | 0.97 | 68 | 0.64 | 69 | 1.38 | 69 | 1.71 | 70 | 2.19 |
|  | (0.15; 0.63)** |  | / |  | (-0.47; 2.41) |  | (-0.80; 2.08) |  | (-0.07; 2.82) |  | (0.25; 3.17)* |  | (0.72; 3.66)** |

^(1)^ For each dimension, the modelled outcome is the average of the top-box score percentages for all questions within that dimension, except for the two questions of the dimension global which are modelled separately. Questions and dimensions of the Flemish Patient Survey (FPS) are copyright protected. For further information on the usage of the FPS: contact [info@vlaamspatientenplatform.be](mailto:info@vlaamspatientenplatform.be)

^(2)^ Linear estimate (with 95% confidence interval), i.e. the yearly change in percentage top-box scores.

^(3)^ Estimate (with 95% confidence interval) treating year as categorical variable, i.e. the change in percentage top-box scores for a given year, relative to the reference year (2014).

* Statistically significant at an alpha level of 0.05. ** Statistically significant at an alpha level of 0.01.
