# Supplementary material for: Six years of measuring patient experiences in Belgium: Limited improvement and lack of association with improvement strategies
Source: PLoS One. 2020 Nov 3;15(11):e0241408. doi: 10.1371/journal.pone.0241408 (PMC7608918; doi:10.1371/journal.pone.0241408)
Supplement: S2 Table — (DOCX) [file pone.0241408.s002.docx]

**S2 Table.** **Associations between quality improvement strategies and average top-box scores of the 8 patient experience dimensions in 2019.**

| **Surveyed quality improvement strategy** | **Dimension of patient experience^(1)^** | **β^(2)^** | **(95% CI)** |
| --- | --- | --- | --- |
| FPS feedback to clinicians | Preparing for hospital stay | -2.32 | (-6.96; 2.31) |
|  | Information about condition | -2.87 | (-7.77; 2.04) |
|  | Information about treatment and procedures | -2.95 | (-6.44; 0.54) |
|  | Dealing with patients and collaboration between healthcare providers | -1.79 | (-5.35; 1.77) |
|  | Privacy | 0.06 | (-3.75; 3.87) |
|  | Safe care | -1.38 | (-6.90; 4.14) |
|  | Pain management | -3.80 | (-8.18; 0.58) |
|  | Discharge | -1.81 | (-3.53; -0.08)* |
| Nursing ward interventions | Preparing for hospital stay | -0.89 | (-5.22; 3.45) |
|  | Information about condition | 4.73 | (0.36; 9.10)* |
|  | Information about treatment and procedures | 2.10 | (-1.18; 5.38) |
|  | Dealing with patients and collaboration between healthcare providers | 4.27 | (1.22; 7.32)** |
|  | Privacy | 4.70 | (1.50; 7.90)** |
|  | Safe care | 3.10 | (-1.93; 8.13) |
|  | Pain management | 2.39 | (-1.76; 6.53) |
|  | Discharge | 0.38 | (-1.30; 2.06) |
| Hospital wide intervention | Preparing for hospital stay | 1.37 | (-2.95; 5.70) |
|  | Information about condition | 2.72 | (-1.82; 7.26) |
|  | Information about treatment and procedures | 1.44 | (-1.88; 4.76) |
|  | Dealing with patients and collaboration between healthcare providers | 3.55 | (0.41; 6.70)* |
|  | Privacy | 3.90 | (0.60; 7.21)* |
|  | Safe care | 3.60 | (-1.40; 8.60) |
|  | Pain management | 0.98 | (-3.23; 5.18) |
|  | Discharge | 0.19 | (-1.49; 1.87) |
| Board sets strategy | Preparing for hospital stay | -0.84 | (-4.71; 3.03) |
|  | Information about condition | 1.49 | (-2.61; 5.58) |
|  | Information about treatment and procedures | 1.11 | (-1.86; 4.08) |
|  | Dealing with patients and collaboration between healthcare providers | 1.08 | (-1.88; 4.04) |
|  | Privacy | 2.03 | (-1.05; 5.12) |
|  | Safe care | 2.96 | (-1.52; 7.44) |
|  | Pain management | -1.58 | (-5.30; 2.15) |
|  | Discharge | 0.36 | (-1.14; 1.86) |
| FPS targets | Preparing for hospital stay | 0.75 | (-2.63; 4.13) |
|  | Information about condition | -1.41 | (-4.98; 2.17) |
|  | Information about treatment and procedures | -1.45 | (-4.02; 1.12) |
|  | Dealing with patients and collaboration between healthcare providers | -0.27 | (-2.87; 2.33) |
|  | Privacy | 0.08 | (-2.67; 2.84) |
|  | Safe care | 2.20 | (-1.74; 6.13) |
|  | Pain management | -1.27 | (-4.53; 1.98) |
|  | Discharge | -0.84 | (-2.12; 0.44) |
| Hospital wide education | Preparing for hospital stay | -0.09 | (-3.26; 3.08) |
|  | Information about condition | 3.02 | (-0.21; 6.25) |
|  | Information about treatment and procedures | 1.61 | (-0.78; 4.00) |
|  | Dealing with patients and collaboration between healthcare providers | 2.29 | (-0.03; 4.61) |
|  | Privacy | 2.27 | (-0.20; 4.74) |
|  | Safe care | 1.54 | (-2.17; 5.25) |
|  | Pain management | 1.86 | (-1.15; 4.87) |
|  | Discharge | 0.37 | (-0.85; 1.59) |
| Discharge info on admission | Preparing for hospital stay | -1.12 | (-4.27; 2.03) |
|  | Information about condition | 2.77 | (-0.48; 6.02) |
|  | Information about treatment and procedures | 2.12 | (-0.23; 4.47) |
|  | Dealing with patients and collaboration between healthcare providers | 0.53 | (-1.90; 2.96) |
|  | Privacy | -0.27 | (-2.84; 2.30) |
|  | Safe care | 0.14 | (-3.59; 3.88) |
|  | Pain management | 0.16 | (-2.91; 3.23) |
|  | Discharge | 0.23 | (-0.99; 1.45) |
| Nursing rounds | Preparing for hospital stay | -1.03 | (-4.12; 2.06) |
|  | Information about condition | 0.02 | (-3.28; 3.33) |
|  | Information about treatment and procedures | -0.88 | (-3.26; 1.50) |
|  | Dealing with patients and collaboration between healthcare providers | -0.22 | (-2.60; 2.17) |
|  | Privacy | 1.22 | (-1.28; 3.71) |
|  | Safe care | 1.65 | (-1.98; 5.28) |
|  | Pain management | -0.27 | (-3.28; 2.74) |
|  | Discharge | -0.52 | (-1.71; 0.67) |
| HR policy | Preparing for hospital stay | -1.15 | (-4.23; 1.94) |
|  | Information about condition | -0.43 | (-3.72; 2.87) |
|  | Information about treatment and procedures | -0.93 | (-3.31; 1.44) |
|  | Dealing with patients and collaboration between healthcare providers | 0.06 | (-2.33; 2.44) |
|  | Privacy | -0.20 | (-2.72; 2.32) |
|  | Safe care | 1.29 | (-2.35; 4.93) |
|  | Pain management | 1.80 | (-1.16; 4.75) |
|  | Discharge | 0.34 | (-0.85; 1.54) |
| Proactive discharge calls | Preparing for hospital stay | 1.48 | (-1.63; 4.58) |
|  | Information about condition | 3.51 | (0.37; 6.66)* |
|  | Information about treatment and procedures | 1.60 | (-0.76; 3.97) |
|  | Dealing with patients and collaboration between healthcare providers | 1.56 | (-0.80; 3.92) |
|  | Privacy | 1.09 | (-1.43; 3.62) |
|  | Safe care | 3.25 | (-0.31; 6.80) |
|  | Pain management | -0.41 | (-3.45; 2.63) |
|  | Discharge | -0.37 | (-1.58; 0.84) |
| Bedside briefing | Preparing for hospital stay | -0.74 | (-3.90; 2.42) |
|  | Information about condition | 1.13 | (-2.22; 4.48) |
|  | Information about treatment and procedures | 0.01 | (-2.44; 2.45) |
|  | Dealing with patients and collaboration between healthcare providers | -0.94 | (-3.35; 1.48) |
|  | Privacy | -0.58 | (-3.14; 1.99) |
|  | Safe care | 1.94 | (-1.75; 5.63) |
|  | Pain management | -0.81 | (-3.86; 2.25) |
|  | Discharge | -0.76 | (-1.96; 0.44) |
| Social media follow-up | Preparing for hospital stay | 1.34 | (-2.22; 4.90) |
|  | Information about condition | -2.24 | (-5.99; 1.50) |
|  | Information about treatment and procedures | -1.51 | (-4.23; 1.21) |
|  | Dealing with patients and collaboration between healthcare providers | 0.70 | (-2.05; 3.44) |
|  | Privacy | -1.46 | (-4.33; 1.41) |
|  | Safe care | -2.74 | (-6.88; 1.40) |
|  | Pain management | 1.43 | (-2.01; 4.87) |
|  | Discharge | 0.10 | (-1.28; 1.49) |
| FPS nursing ward rewards | Preparing for hospital stay | -1.06 | (-4.52; 2.40) |
|  | Information about condition | -0.60 | (-4.29; 3.09) |
|  | Information about treatment and procedures | -0.95 | (-3.62; 1.71) |
|  | Dealing with patients and collaboration between healthcare providers | -0.59 | (-3.26; 2.07) |
|  | Privacy | 0.43 | (-2.38; 3.25) |
|  | Safe care | -0.16 | (-4.26; 3.95) |
|  | Pain management | -2.13 | (-5.43; 1.17) |
|  | Discharge | -0.45 | (-1.79; 0.89) |
| Multidisciplinary discharge | Preparing for hospital stay | 1.58 | (-2.27; 5.43) |
|  | Information about condition | 0.97 | (-3.14; 5.09) |
|  | Information about treatment and procedures | 0.98 | (-2.00; 3.95) |
|  | Dealing with patients and collaboration between healthcare providers | 0.75 | (-2.22; 3.72) |
|  | Privacy | -0.51 | (-3.65; 2.64) |
|  | Safe care | -2.57 | (-7.07; 1.94) |
|  | Pain management | 0.80 | (-2.95; 4.55) |
|  | Discharge | 0.97 | (-0.50; 2.44) |
| External consultants | Preparing for hospital stay | -6.20 | (-11.76; -0.65)* |
|  | Information about condition | 3.30 | (-2.88; 9.48) |
|  | Information about treatment and procedures | 0.92 | (-3.61; 5.45) |
|  | Dealing with patients and collaboration between healthcare providers | -1.41 | (-5.91; 3.10) |
|  | Privacy | 1.54 | (-3.22; 6.30) |
|  | Safe care | 6.38 | (-0.27; 13.04) |
|  | Pain management | -4.71 | (-10.22; 0.80) |
|  | Discharge | -2.78 | (-4.88; -0.67)* |

^(1)^ Questions and dimensions of the Flemish Patient Survey (FPS) are copyright protected. For further information on the usage of the FPS: contact [info@vlaamspatientenplatform.be](mailto:info@vlaamspatientenplatform.be)

^(2)^ The difference (with 95% confidence interval) in percentage top-box scores between hospitals with and without the improvement strategy.

* Statistically significant at an alpha level of 0.05. ** Statistically significant at an alpha level of 0.01.

None of the estimates were significant after Bonferroni correction.
