# Supplementary material for: Six years of measuring patient experiences in Belgium: Limited improvement and lack of association with improvement strategies
Source: PLoS One. 2020 Nov 3;15(11):e0241408. doi: 10.1371/journal.pone.0241408 (PMC7608918; doi:10.1371/journal.pone.0241408)

**S1 File. Associations between quality improvement strategies and time trends in average top-box scores of the 8 patient experience dimensions.**

The plotted time trends are the predictions from multilevel regression models containing a binary indicator for strategy implementation, a linear variable for year, and an interaction between these variables. The p-value represents the significance of the interaction term and indicates whether time trends are significantly different between hospitals with and without a given strategy.


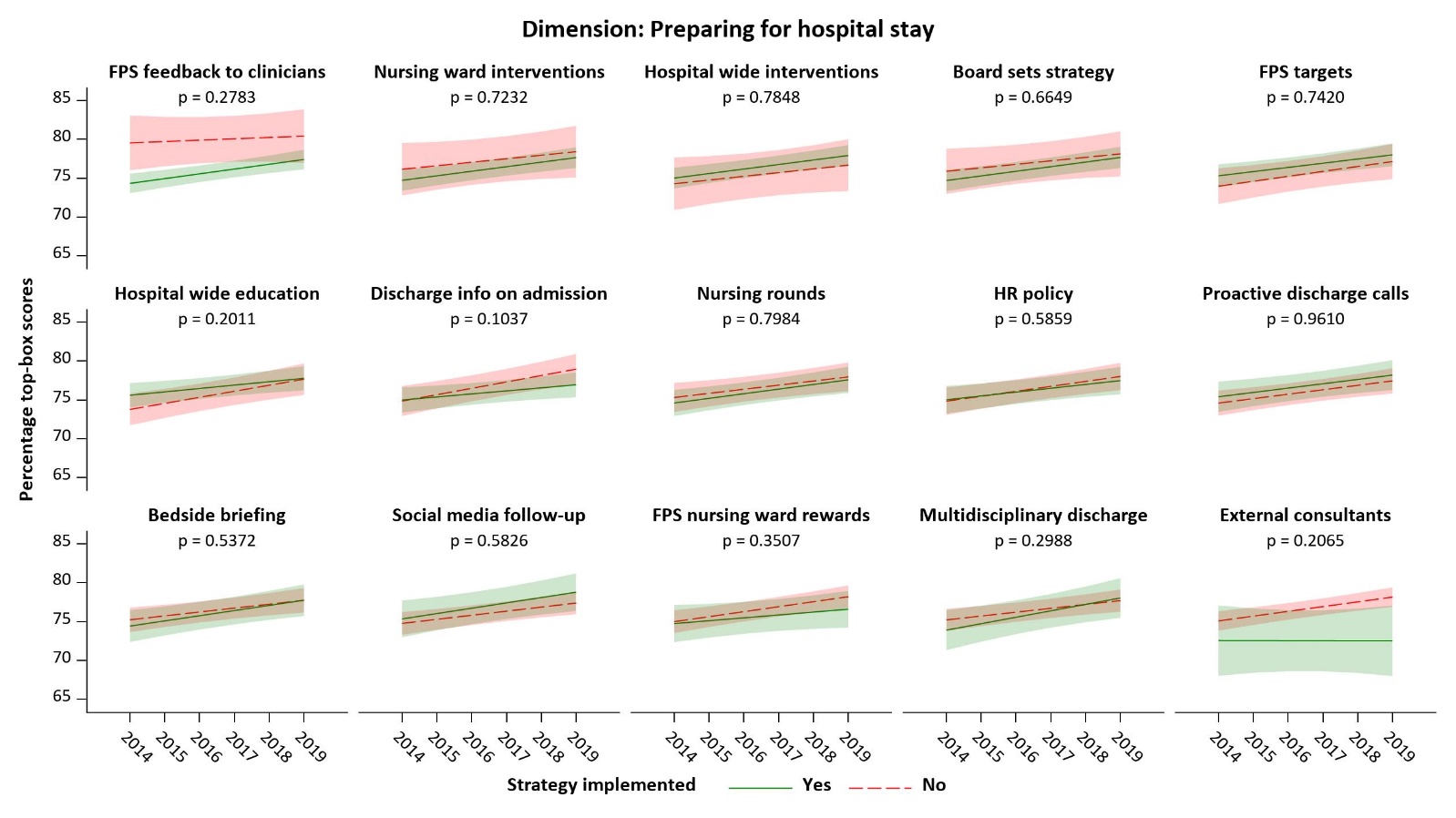


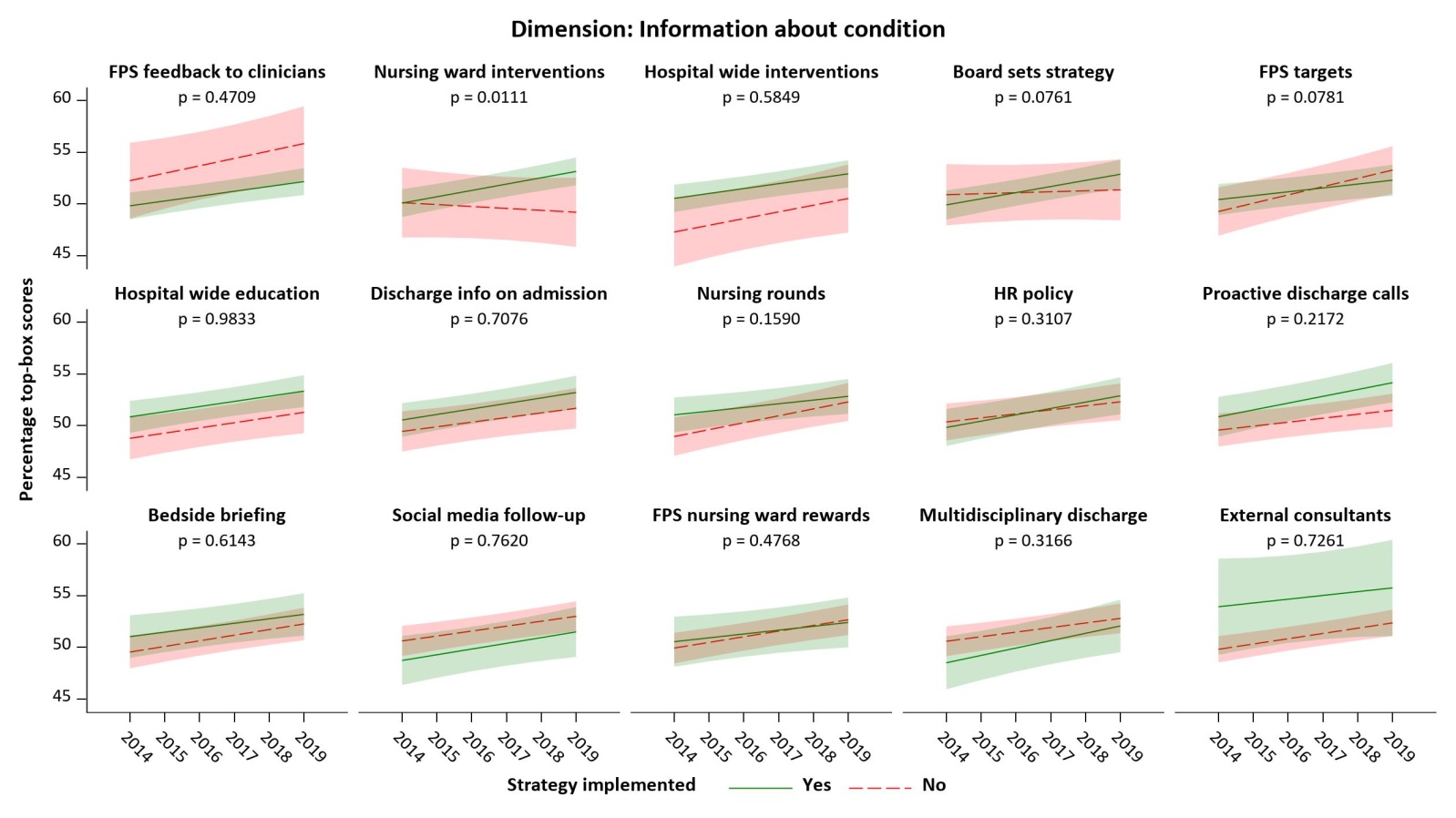


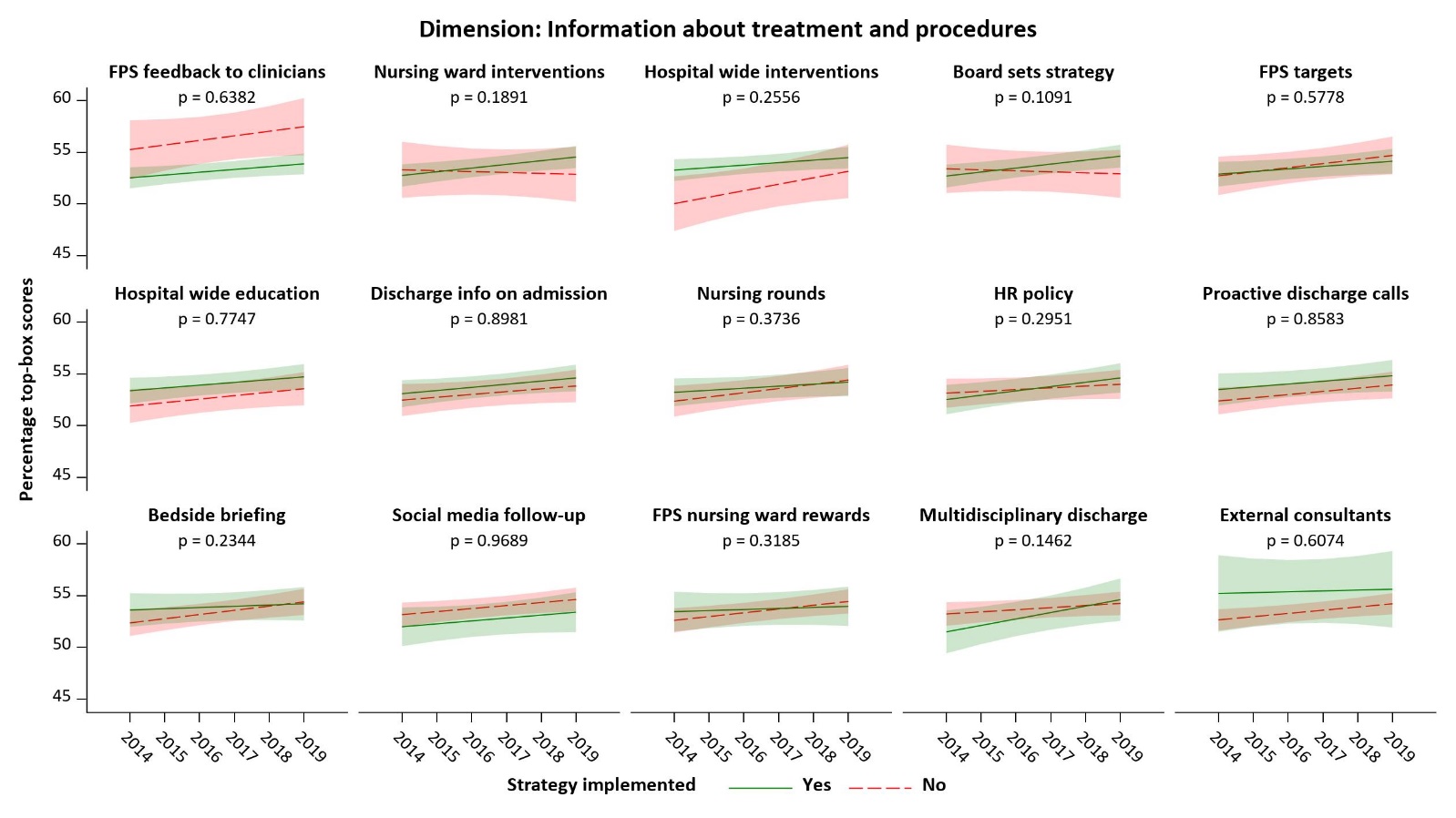


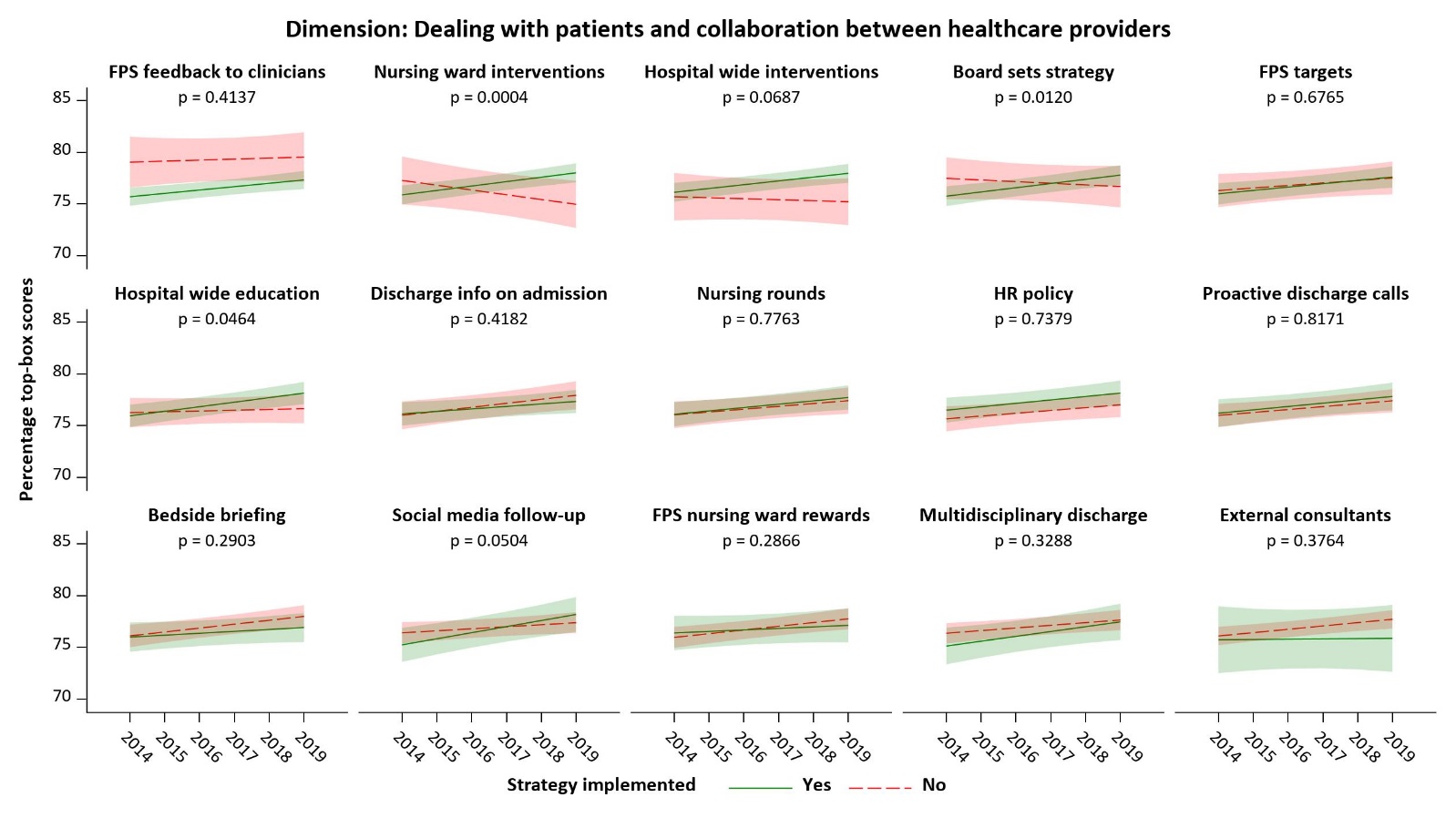


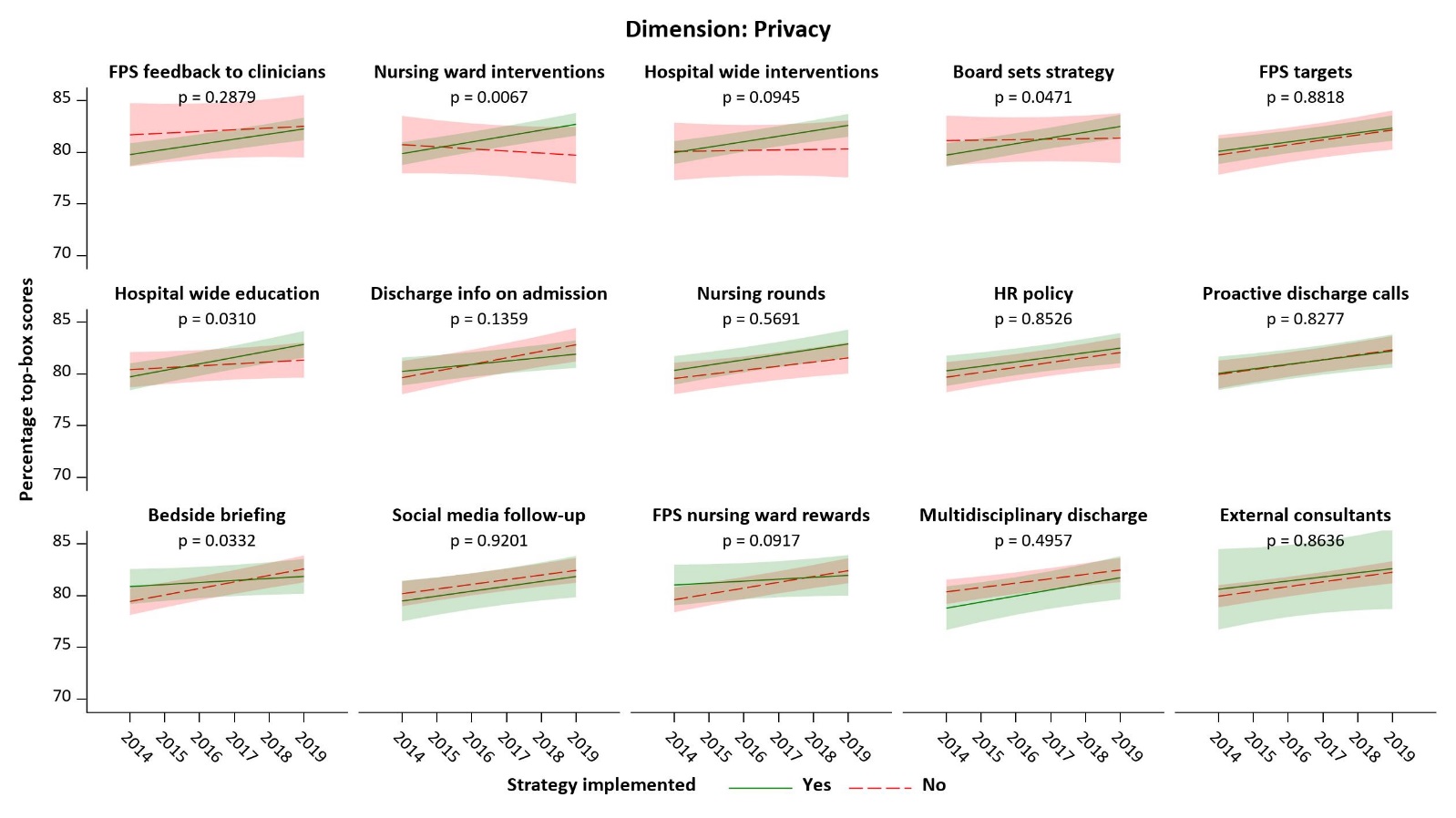


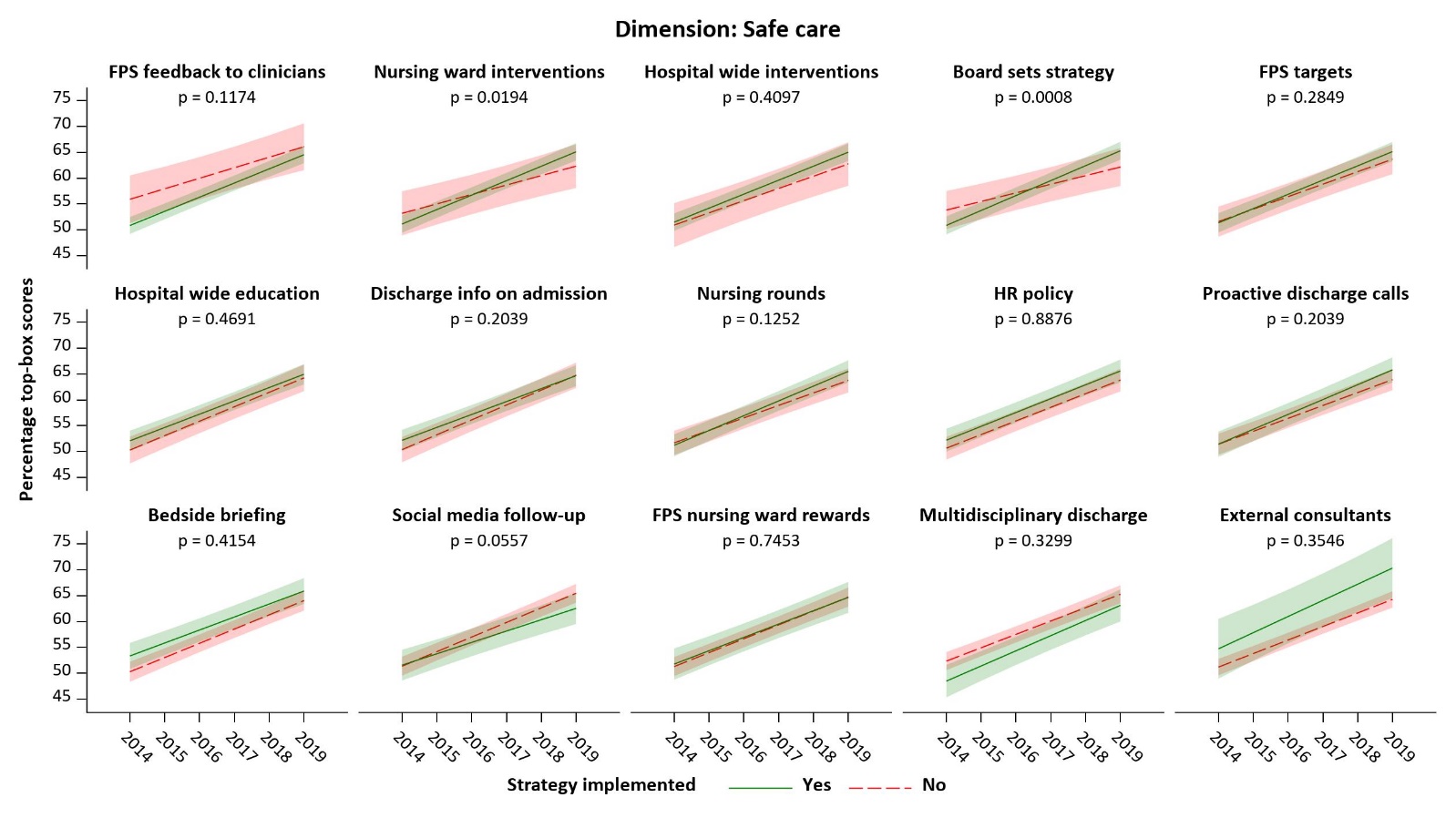


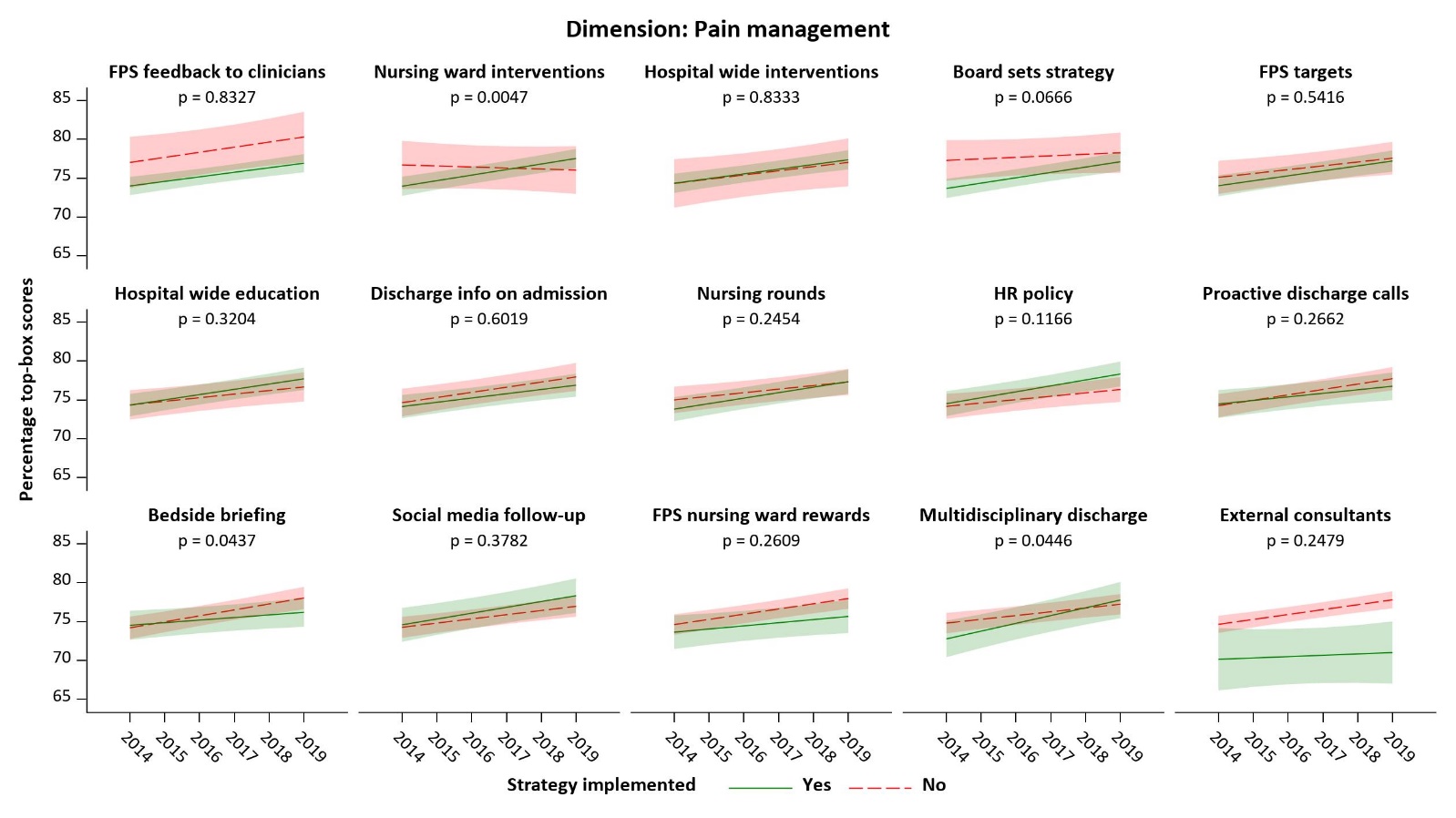


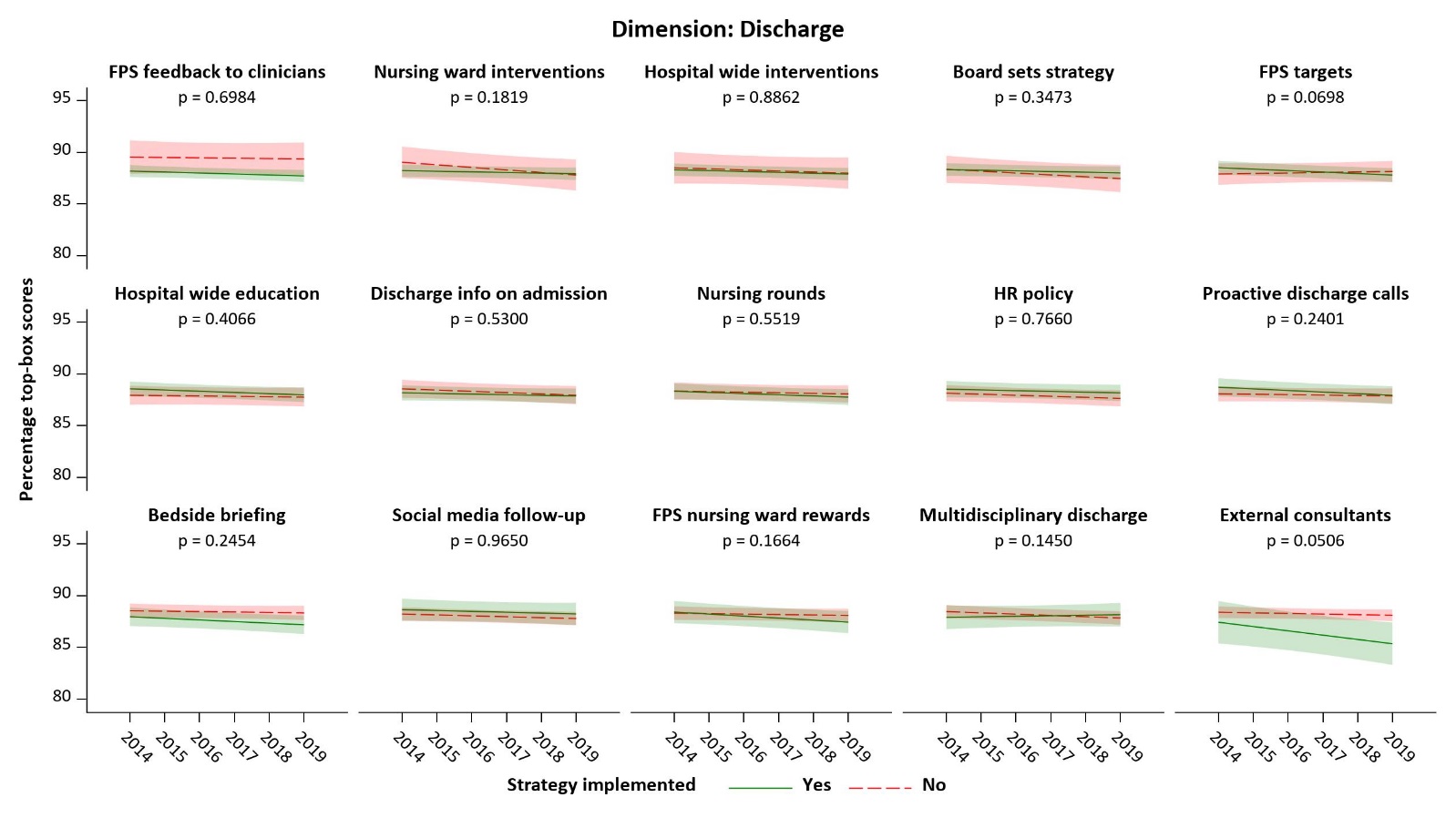

Supplement: S1 File — (DOCX) [file pone.0241408.s003.docx]
