# Supplementary material for: Six years of measuring patient experiences in Belgium: Limited improvement and lack of association with improvement strategies
Source: PLoS One. 2020 Nov 3;15(11):e0241408. doi: 10.1371/journal.pone.0241408 (PMC7608918; doi:10.1371/journal.pone.0241408)
Supplement: Attachment — Submitted filename: Response to Reviewers.docx [file pone.0241408.s005.docx]

**Response to reviewers’ comments**

PLOS ONE Minor Revision

**‘Six years of measuring patient experiences in Belgium: limited improvement and lack of association with improvement strategies.’**

PONE-D-20-04886

Dear Sir,

Dear Madam,

We are once again incredibly grateful for the thorough synthesis and review of our manuscript by the PLOS ONE editor and external peer reviewers. We are glad our previous changes have overall been well received and feel the additional comments made in this review have merit and will add to an improved manuscript. Outlined below you will find our point-by-point response to all comments to demonstrate how we took them to heart and adjusted our manuscript. The line numbers refer to the revised manuscript document without tracked changes.

Academic Editor’s Comments

1. Please revise as per reviewer one’s comment.

*See reply below.*

2. As per my suggestion in the previous comments, please provide additional information on your recommendations, particularly on those interventions that had a positive impact on patient experience – “Nursing ward interventions” and “Hospital wide education.”

*We added additional information on recommendations for practice in lines 328 to 336 and stated the following from line 332: “The examples provided by some participating hospitals such as e.g. mealtimes between staff and patients or the development of hospital-wide courses, suggest a large variety of ways to execute strategies. We thus encourage hospitals to share and learn from both their positive and negative experiences. By focusing on both nursing ward interventions and hospital wide education, a high visibility for the patient as well as a widespread reach of all healthcare staff can be ensured.”*

*We hope this information can be applied by hospitals reading this manuscript that are aiming to improve their patients’ experiences.*

3. Also, as recommended by reviewer one, please add this information on the positive interventions to the abstract.

*We added information on the positive associations between improvement strategies and patient experiences both in the results and conclusion section of the abstract. As such, we wrote the following in line 59: “Still, positive associations were discovered between the strategies ‘nursing ward interventions’ and ‘hospital wide education’ and recommendation of the hospital.” Additionally, in line 62, we wrote: “Hospitals report to have invested in patient experience improvement strategies but positive associations between such strategies and FPS scores are weak, although there is potential in further exploring nursing ward interventions and hospital wide education.”*

4. Revise the final sentence of the abstract to reflect a more scholarly approach.

*We altered the final sentence in our abstract to “Hospitals should continue their efforts to improve the patient’s experience, but with a more targeted approach, taking the lessons learned on the efficacy of strategies into consideration.” (line 65). As such, we hope to have provided a more nuanced conclusion that takes the improvements already made into account, but also highlights the fact that most strategies have not been associated with improvements in patient experience scores. A further focus on strategies that could potentially benefit the patient’s experience is therefore required.*

Reviewers' comments:

REVIEWER #1

1. The only comment I would now add is that more could be made of the two strategies that appear to be associated with improvement, i.e. ‘Nursing ward interventions’ and ‘Hospital wide education’ as these are the two practical things that a reader could take from the paper. I would make two suggestions. First, somewhere in the discussion (or an appendix if necessary), the paper should describe what these actually are, to the extent that the information is available.

*As we mentioned in our previous review, as well as in our limitation section (line 351) of our discussion, detailed information on when, how and on what ward the hospitals chose to implement their employed strategies, is unavailable for the majority of participating hospitals. Nevertheless, we understand the need for more detailed information on the surveyed strategies, in particular those with potential benefit towards the patient’s experience. We therefore contacted the quality managers of three participating hospitals that all indicated they employed both nursing ward interventions and hospital wide education and asked them to elaborate on how they employed these strategies. While exact details could not be provided, they could give examples of interventions they had implemented, which add to the understanding of what that particular strategy entails. As such, we added more information in the description section of Table 1 (line 234) for both nursing ward interventions, hospital wide interventions and hospital wide education. We provided extra information in the discussion section as well in line 332: “The examples provided by some participating hospitals such as e.g. mealtimes between staff and patients or the development of hospital-wide courses, suggest a large variety of ways to execute strategies. We thus encourage hospitals to share and learn from both their positive and negative experiences. By focusing on both nursing ward interventions and hospital wide education, a high visibility for the patient as well as a widespread reach of all healthcare staff can be ensured.”*

*In addition, we put more emphasis on the potential benefits of nursing ward interventions and hospital wide education throughout the discussion section. First, we added lines 315 to 318: “What’s more, both nursing ward interventions and hospital wide education were found to be associated with better 2019 FPS results. Additionally, nursing ward interventions in particular were positively associated with improved global patient experiences over time.” Second, we wrote lines 321-324: “Despite the positive associations between both nursing ward interventions and hospital wide education and 2019 FPS results and the positive relationship between nursing ward interventions and recommendation of the hospital, improvement strategies were overall not or only weakly associated with patient experience ratings.” Last, we made some considerations for practice in lines 328-336: “Considering its potential, further research into the benefits of nursing ward interventions or a hospital-wide educational program is advised. By researching the evidence-base on the interventions that have shown most promise, we hope future healthcare policy and practice might be altered towards a more unified care, instead of the wide spectrum of sometimes ineffective interventions currently implemented. The examples provided by some participating hospitals such as e.g. mealtimes between staff and patients or the development of hospital-wide courses, suggest a large variety of ways to execute strategies. We thus encourage hospitals to share and learn from both their positive and negative experiences. By focusing on both nursing ward interventions and hospital wide education, a high visibility for the patient as well as a widespread reach of all healthcare staff can be ensured.”*

*By the best of our abilities, we thus hope to have provided you with a deeper understanding of how the surveyed strategies could be implemented in practice.*

2. Second, I would recommend that these two findings (almost the only positive findings in the paper) should be included in the abstract.

*We added information on the positive associations between improvement strategies and patient experiences both in the results and conclusion section of the abstract. As such, we wrote the following in line 59: “Still, positive associations were discovered between the strategies ‘nursing ward interventions’ and ‘hospital wide education’ and recommendation of the hospital.” Additionally, in line 62, we wrote: “Hospitals report to have invested in patient experience improvement strategies but positive associations between such strategies and FPS scores are weak, although there is potential in further exploring nursing ward interventions and hospital wide education.”*

3. Personally, I don’t like the last sentence of the abstract which is expressed in rather unscientific language. I also don’t know if it’s overstated – e.g. in relation to the world literature on quality improvement, is a 5% increase in top scoring hospitals in five years that bad? This could also be addressed in the discussion.

*We altered the final sentence in our abstract to “Hospitals should continue their efforts to improve the patient’s experience, but with a more targeted approach, taking the lessons learned on the efficacy of strategies into consideration.” (line 65). As such, we hope to have provided a more nuanced conclusion that takes the improvements already made into account, but also highlights the fact that most strategies have not been associated with improvements in patient experience scores. A further focus on strategies that could potentially benefit the patient’s experience is therefore required.*

*Lastly, to further nuance the improvements made by the participating hospitals, we removed the comment ‘yet small’ (talking about significant improvement) from the results section (line 53) of the abstract. As you rightly pointed out, a 5% increase over the course of 5 years is still a commendable achievement. We also highlight this in line 296 of the discussion of the manuscript: “The overall improvement, strongest in most recent years, is commendable, yet small.” Still, like our discussion continues, we believe there is still room for improvement, considering the achievements made in other countries and considering the fact most of the surveyed strategies had no impact on the patient’s experience.*

We would like to thank you again for considering our manuscript for publication in your journal.

Yours sincerely,

Astrid Van Wilder
